# Coexistence of Right Nonrecurrent Nerve and Bifurcated Recurrent Laryngeal Nerve Pointed by Zuckerkandl's Tubercle

**DOI:** 10.7759/cureus.1078

**Published:** 2017-03-05

**Authors:** Emin Gurleyik, Sami Dogan, Fuat Cetin

**Affiliations:** 1 Department of Surgery, Duzce University Medical Faculty

**Keywords:** thyroid, surgery, anatomical variation, inferior laryngeal nerve

## Abstract

The recurrent laryngeal nerve (RLN) has many anatomical variations and various relations with adjacent structures. Identification and total exposure of the cervical part of the RLN was performed during operations on the thyroid gland. An extremely rare anatomical variation of the nerve was encountered during the surgical procedure. Coexistence of both right RLN and non-RLN was observed in one patient surgically treated with total thyroidectomy. We first exposed the right RLN with an extralaryngeal terminal bifurcation at its usual position. Thereafter, we also identified an ipsilateral non-RLN joining the anterior branch of the RLN just before laryngeal entry. A Zuckerkandl's tubercle has pointed out the junction of the two nerves. In this period, the incidence of coexistence of non-RLN and RLN was 0.2% in our series. A non-recurrent course is a rare anatomical variation of the inferior laryngeal nerve. The coexistence of both non-RLN and RLN is an extremely rare anatomical finding which should be taken into account during thyroid surgery.

## Introduction

In terms of complications, the recurrent laryngeal nerve (RLN) is the most important structure in thyroid surgery. Full knowledge of the anatomy of the RLN including all its variations is mandatory for complication-free thyroidal surgery. Anatomical variations of the RLN which increase injury rate threaten the safety of thyroid surgery. A non-recurrent course of the nerve is a rare variation which is a significant risk for vocal cord palsy. A non-RLN is always found on the right side. This nerve originating from the cervical part of the vagus enters the larynx after a short course without recurrence under the subclavian artery. Sometimes, a secondary variation of a rare variation can occur in the same patient.

In this manuscript, we report an extremely rare case of anatomical variation of the RLN. Informed consent was obtained from all patients for this study.      

## Case presentation

**Figure 1:**
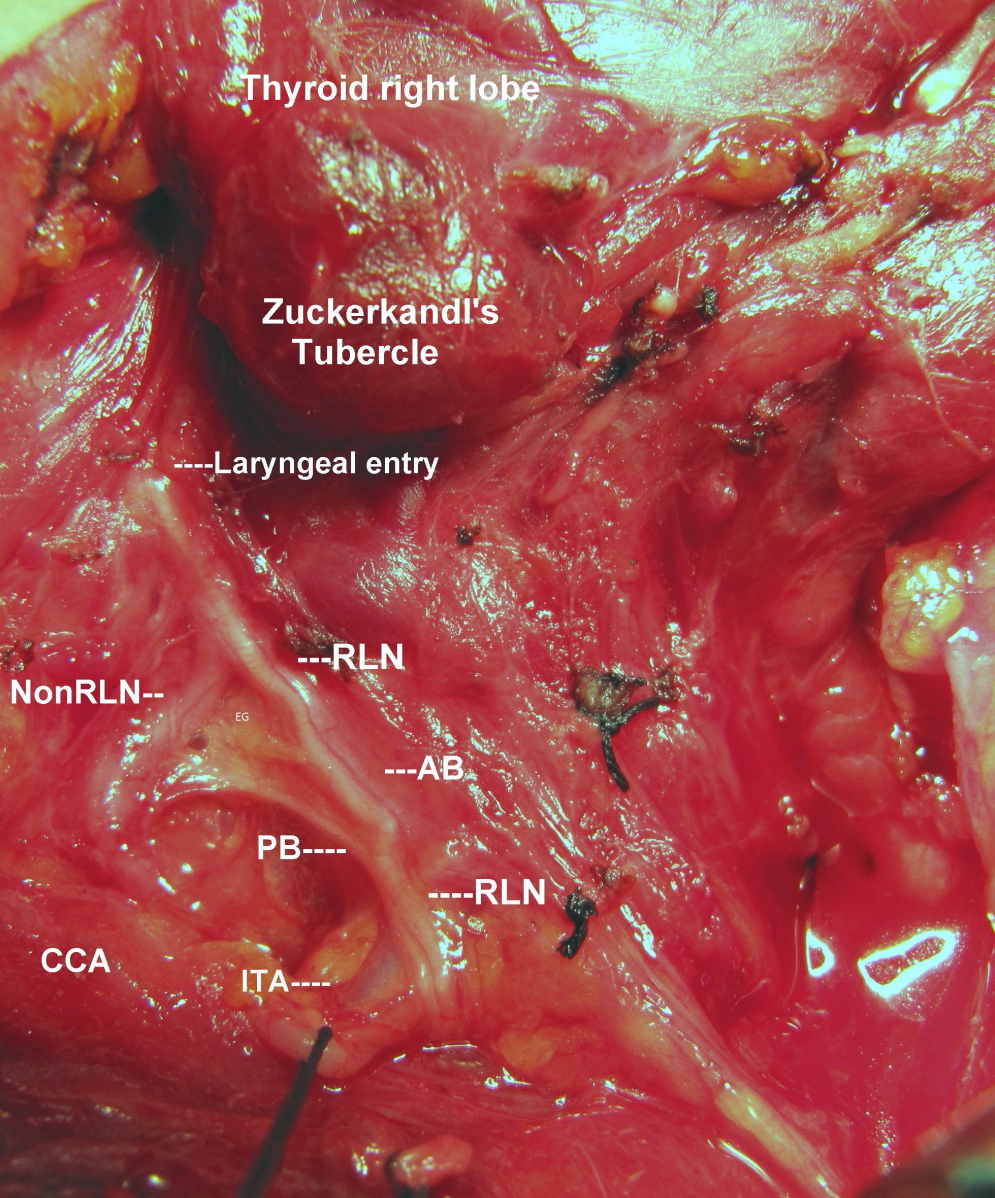
Operative photograph showing RLN and non-RLN The cervical part of bifurcated right recurrent laryngeal nerve (RLN) is fully exposed at its usual position until laryngeal entry. A secondary non-RLN which has a transversal course joins to anterior branch of the RLN. AB: Anterior branch. PB: Posterior branch. ITA: Inferior thyroid artery. CCA: Common carotid artery.

In the last six years, we performed 512 (total or right hemi) thyroidectomies in our department of surgery. All RLNs were totally exposed along their cervical courses. The patient presented in this report was suffering from hyperthyroidism. Suppressed serum thyroid stimulating hormone (TSH) and high free thyroxine (free T4) levels were determined by biochemical analysis. Ultrasound and nuclear scans of the thyroid gland revealed a 13 mm solid and hot nodule in the right lobe, and a 23 mm solid and hyperactive nodule in the left. The diagnosis was toxic multinodular goiter. Total thyroidectomy was the appropriate surgical treatment for this patient. We totally exposed the cervical segments of both RLNs. On the right side, the RLN was exposed at its usual position and followed until laryngeal entry. The nerve had an extralaryngeal bifurcation with larger anterior and thinner posterior branches that separately entered the larynx (Figure 1). We discovered a second inferior laryngeal nerve which entered the surgical area from the posterior side of the carotid artery and traversed directly to the larynx with a nonrecurrent short horizontal course. The non-RLN joined the anterior branch of ipsilateral RLN before laryngeal entry. Both the RLN and non-RLN had typical red stripes on their anterior surfaces, the "vasa nervorum" (Figure 1).

The incidence of coexistence of RLN and non-RLN was 0.2% during this period. The right lobe of the thyroid gland had a posterior extension - the Zuckerkandl's tubercle (ZT). The tubercle seemed to point out the junction of the RLN and non-RLN just before laryngeal entry (Figure 1). The postoperative period was uneventful, and the patient was discharged without operative complications.     

## Discussion

In our patient, the right RLN had an anatomical variation, being divided into two branches. This was an extralaryngeal bifurcation with larger anterior and thinner posterior branches. Bifurcation of the RLN before laryngeal entry has been reported to be a common variation [1-2]. It may further increase the risk of nerve injury during thyroid surgery. Taking into account this common situation, all neural structures must be protected to preserve the motor function of the laryngeal musculature.

The right lobe of the thyroid in our patient had a posterior extension, the ZT. The surgical importance of the tubercle depends mainly on two points: the completeness of thyroidectomy and its relation with the RLN. Excision of the tubercle is required for complete resection of the thyroid tissue. On the other hand, its excision is technically difficult because of its close relationship with the RLN. It has been reported that the ZT is a common anatomical structure. The RLN has a cervical course generally medial and uncommonly lateral to the tubercle [3]. In our case, the ZT pointed out the nerve like an arrow head. 

The RLN has many anatomical variations and various relationships with adjacent structures that complicate thyroid surgery. One of these variations is the nonrecurrent course of the nerve. The incidence of non-RLN has been reported to be between 0.3% and 1.3% in previous series [4-7]. Therefore, the nonrecurrent course is a rare anatomical variation of the inferior laryngeal nerve.

A literature search reveals an extremely rare anatomical variation of the RLN. Obaid, et al. [8] have reported that only 14 cases of coexisting RLN and non-RLN were identified in the literature. Our patient is an example of this variation, which we encountered during total thyroidectomy. A photograph of the surgical area showed similar sized RLN and non-RLN after visual identification and full exposure of neural structures. Medline has classified a few reported cases [8-10]. During our study period, 0.2% incidence of the coexistence of RLN and non-RLN indicates the extremely rare presence of this variation. Previous reports have also mentioned that this occurrence is very rare with incidence lying between 0.03% and 0.2% [4-6]. We think that the incidence of this variation may be higher than the reported numbers. Once a thyroid surgeon identifies and exposes the RLN at its usual position, he does not generally search for a second laryngeal nerve. In our patient, a secondary non-RLN was an incidental finding during surgical dissection.  

## Conclusions

The inferior laryngeal nerve has many common, uncommon, or rare anatomical variations. Sometimes, these variations synchronously occur in the same patient like in ours. A nonrecurrent course is a rare anatomical variation of the inferior laryngeal nerve. Coexistence of both non-RLN and RLN on the right side in the same patient is an extremely rare anatomical finding which should be taken into account during thyroid surgery. 
